# Genetic variation at a splicing branch point in intron 7 of *STK11*: a rare variant decreasing its expression in a Chinese family with Peutz–Jeghers syndrome

**DOI:** 10.1186/s12957-024-03475-6

**Published:** 2024-07-30

**Authors:** Xiufang Wang, Yuanyuan Li, Jingqiong Zhang, Chao Liu, Aiping Deng, Juyi Li

**Affiliations:** 1 Department of Pain, Tongji Medical College, The Central Hospital of Wuhan, Huazhong University of Science and Technology, Wuhan, Hubei China; 2 Department of Pharmacy, Tongji Medical College, The Central Hospital of Wuhan, Huazhong University of Science and Technology, Wuhan, Hubei China; 3 Department of Oncology, Tongji Medical College, The Central Hospital of Wuhan, Huazhong University of Science and Technology, Wuhan, Hubei China; 4 Hubei Key Laboratory of Diabetes and Angiopathy, Hubei University of Science and Technology, Xianning, 437000 Hubei China

**Keywords:** *STK11*, Peutz–Jeghers syndrome, Gastric-type adenocarcinoma of the cervix, Genetic testing, Variant

## Abstract

**Background:**

Peutz–Jeghers syndrome (PJS), a rare dominantly inherited disease, is primarily characterized by hamartomatous polyps and melanotic macules as well as by an increased risk of cancer. The current study aimed to identify the pathogenic gene and pathogenic mechanism of a proband with PJS, thereby offering precise prevention and treatment strategies for PJS.

**Methods:**

A detailed clinical examination was performed of the proband diagnosed with PJS and her family members. In addition, peripheral venous blood was collected from the family members to extract genomic DNA. The pathogenic genes of the proband were identified using whole-exome sequencing, and the candidate pathogenic variants were verified via Sanger sequencing. Meanwhile, co-segregation tests were performed among six family members. Finally, reverse transcription-polymerase chain reaction (RT-PCR) was performed to assess transcript variants in the peripheral blood cells of patients and non-related healthy controls.

**Results:**

Genetic testing revealed a rare splicing variant c.921-1G > C in *STK11* in the proband and in her sister and nephew, and the variant co-segregated among the affected family members and nonrelated healthy controls. The proband phenotypically presented with a rare gastric-type adenocarcinoma of the cervix. RT-PCR revealed that the *STK11* c.921-1G > C variant could produce two transcripts. Of note, 40 base pairs were deleted in the aberrant transcript between exons 3 and 4, resulting in a frameshift variant and premature termination of the amino acid in exon 6 and ultimately leading to the loss of its functional domain in the STK11 protein. Finally, RT-PCR showed that compared with healthy controls, *STK11* mRNA expression level was < 50% in patients.

**Conclusion:**

The present study results indicated that the rare splicing variant c.921-1G > C in intron 7 of *STK11* may be a pathogenic variant in patients with PJS. However, this variant (in intron 7) may not produce abnormal transcripts (deletion of 40 base pairs between exons 3 and 4), and PJS may be attributed to the decrease in STK11 expression. Therefore, this study emphasized the importance of genetic counseling, pre-symptomatic monitoring, and early complication management in PJS.

**Supplementary Information:**

The online version contains supplementary material available at 10.1186/s12957-024-03475-6.

## Background

Peutz–Jeghers syndrome (PJS), a rare dominantly inherited disease, is mainly characterized by hamartomatous polyps and melanotic macules as well as by an increased risk of other malignant tumors [1]. According to literature, the risk of various tumors of the gastrointestinal tract, breast, ovary, cervix, and testis in patients with PJS is > 10-fold higher than that among healthy individuals [2]. Among female patients, breast cancer, cervical malignant adenoma, gastric-type adenocarcinoma of the cervix (GAS), minimal deviation adenocarcinoma, round tube tumors, and bilateral mucinous ovarian tumors are believed to be associated with PJS [3, 4].

The serine-threonine kinase 11 (*STK11*) gene, also referred to as liver kinase B1 (LKB1) and present in the chromosomal region 19p13.3, encodes the LKB1 protein [5]. Previous studies have shown that an LKB1 functional deletion variant is the pathogenic cause of PJS [6, 7]. At present, > 200 variants in this gene have been identified in PJS and other sporadic tumors, primarily including splicing site changes, insertion variants, missense variants, nonsense variants, frameshift variants, copy number variations, and even the entire *STK11* deletion [8]. Most variants are located in fragments encoding the active regions of enzymes, resulting in either shortened or missing proteins [9]. *STK11* variants are pathogenic factors not only in PJS but also in several sporadic tumors found throughout the body such as in the colon, stomach, ovary, testis, lung, and other sites. In these sites, tumors are caused by somatic *STK11* or *APC* variants [10–12].

Women with PJS have a higher incidence of GAS. It is an extremely rare malignant tumor that accounts for approximately 2% of all cervical adenocarcinomas [13]. GAS is a newly established entity of cervical mucinous adenocarcinoma, and the distinct morphological features of this entity include a clear and abundant cytoplasm and distinct cell borders and a gastric immunophenotype characterized by HIK1083 or MUC6 expression [14].

Whole-exome sequencing (WES) is a powerful and efficient tool that can obtain high throughput and low-cost whole-exome sequence information [15]. WES can improve the diagnostic yield for monogenic diseases caused by gene variants [16], Therefore, genetic testing is of great significance for PJS diagnosis. In the present study, an *STK11* variant (c.921-1G > C) was detected in a proband with PJS for the first time in China. Moreover, the study revealed the possible pathogenic mechanism of PJS caused by this variant, thereby recommending genetic counseling, pre-symptomatic monitoring, and early complication management in PJS.

## Materials and methods

### Participants

**Fig. 1:**
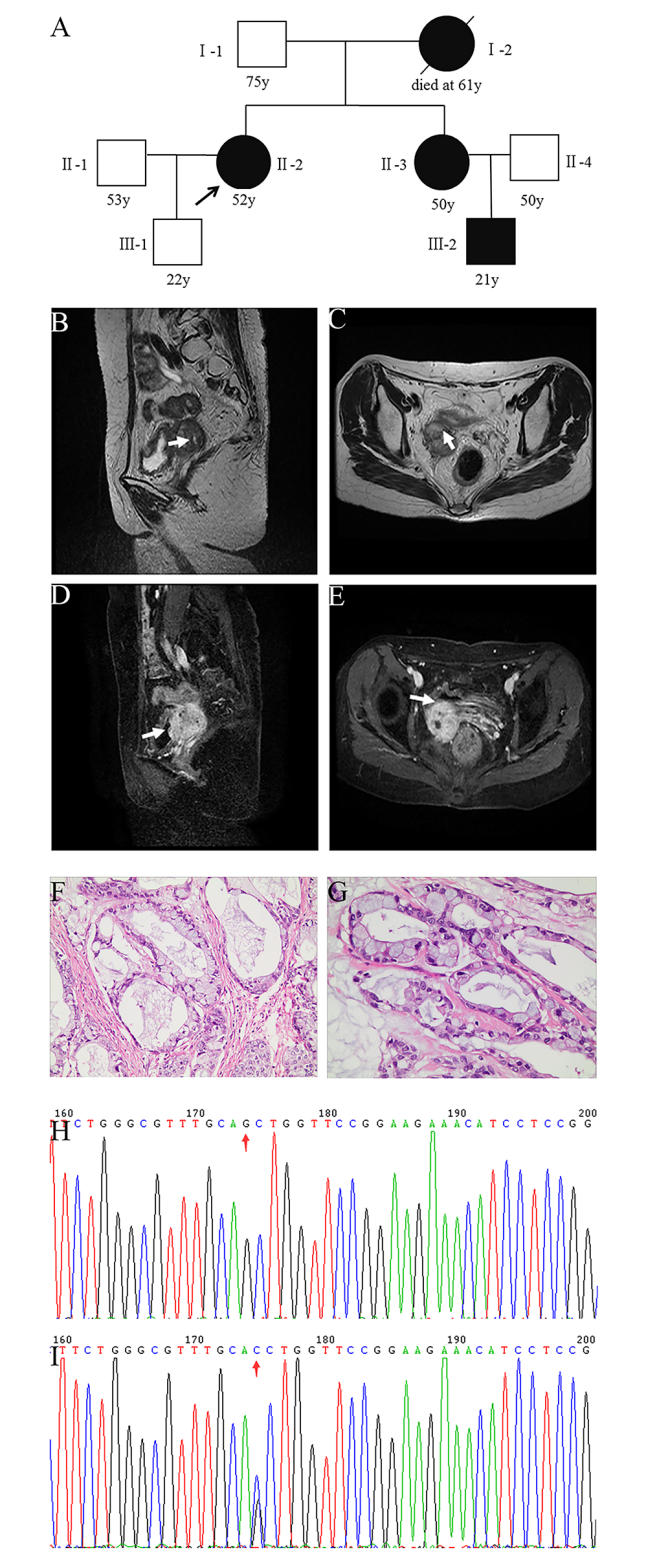
Clinicopathological features of patients with Peutz–Jeghers syndrome (PJS). A: Family pedigree. Squares represent males and circles represent females. An arrow denotes the proband, a solid black box indicates PJS, and a black slash represents death. B–E: pelvic magnetic resonance imaging showing B) sagittal T2WI and C) coronal T2WI. The scan revealed a metabolically active soft tissue mass in the upper right of the vaginal stump (size, approximately 31 × 40 mm); recurrence and invasion of the right ureter were considered. D) Sagittal T2WI and E) coronal T2WI identified a vegetable-patterned mass in the bladder top wall on the right. F–G: Pathological biopsy of the cervix suggested GAS (II-2, 400×). H: *STK11* wild type (c.921-1G). I: *STK11* heterozygote (c.921–1 C)

The current study was approved by the Ethics Committee of the Central Hospital of Wuhan (2020 − 153). The characteristics of all study participants (I-1, II-1, II-2, II-3, III-1, III-2, and two nonrelated healthy controls) and detailed pedigree are shown in Tables 1 and Fig. 1A, respectively. The proband was a 52-year-old woman who had undergone radical trachelectomy, supracervical hysterectomy, and bilateral salpingo-oophorectomy, followed by chemotherapy and radiation, for GAS. The proband, her younger sister (age, 50 years), and nephew (age, 21 years) had developed pigmentation of the lip skin and multiple gastrointestinal polyps during childhood, and they were all clinically diagnosed with PJS. The proband and her family members (I-1, II-1, II-3, III-1, and III-2) were included in this study. Among the other family members, I-1 (age, 75 years), II-1 (age, 53 years), and III-1 (age, 22 years) were included as healthy controls (Fig. 1A). All participants gave their informed consent for participation in this study.

**Table 1:** Physical examination and laboratory review of subjects with PJS

	I-2	II-2	II-3	III-2
Age, y	died at 61	52	50	21
Gender	F	F	F	M
Age of MP	NR	NR	5	6
Reason for visit	Sto	Sto, BO	BO	Sto
Laparotomy Intervention	Ent	EGD	EGD, Ent	EGD, Ent
Tumorigenesis	CC	GAS	GAS	--

Abbreviations: F = female; M = male; Sto = stomach; BO = bowel obstruction; EGD = esophagogastroduodenoscopy; Ent = enteroscopy; MP = mucocutaneous pigmentation (mainly deposited on lips, hands and feet); CC = Cervical cancer; GAS = Gastric-type adenocarcinoma of the uterine cervix; -- = not available; NR = not recorded. II-2: proband; I-2: proband mother; II-3: proband younger sister; III-2: proband nephew

### DNA extraction and WES

Genomic DNA was isolated from the peripheral blood of all participants (six family members and two nonrelated healthy controls) using a DNA extraction kit (Tiangen Biotechnology, Beijing, China) according to the manufacturer’s instructions. Sample preparation and pretreatment were performed using the SureSelect Human All Exon V5 Kit (Agilent Technologies Inc.) for high-throughput sequencing and WES using the Illumina HiSeq 2500 sequencing platform.

### Molecular genetic analyses

The genetic sequence was aligned with the human genome reference sequence (UCSC hg19, GRCh37), and the alignment data were compared and filtered to identify variants, such as single nucleotide polymorphisms (SNPs) and Insertions and deletions. All identified variants were annotated using publicly available databases (1000 Genomes, ExAC, dbSNP, ESP, and gnomAD). In addition, pathogenicity prediction programs (SIFT, PolyPhen2, and MutationTaster) were used to evaluate the potential pathogenicity of the identified nonsynonymous mutations [17]. Finally, Sanger sequencing was used to validate candidate variations and perform segregation analysis.

### Reverse transcription-polymerase chain reaction (RT-PCR) analysis

Total RNA was extracted using the TRIzol reagent (Invitrogen). RT-PCR was performed to detect the transcript variants of *STK11* in patients and two nonrelated healthy controls. The primer sequences for *STK11* analysis using RT-PCR were forward CCAACGTGAAGAAGGAAATTCAACT and reverse CGCCTCTGTGCCGTTCATAC. PCR amplicons were sequenced using Sanger sequencing. GAPDH was used as an internal control.

## Results

### Family characteristics

In all patients, melanin pigmentation was observed. Magnetic resonance imaging of the pelvis (II-2) is shown in Fig. 1B/C/D/E, and the clinicopathological features of the proband are shown in Fig. 1F/G. The mother (I-2) of the proband who died of cervical cancer at the age of 61 years may have had JPS. In addition, her young sister (II-3) and nephew (III-2) were clinically diagnosed with PJS. Furthermore, member II-3 had the same vaginal fluid symptoms as the proband (II-2) and underwent prophylactic hysterectomy and ovarian resection at the age of 48 years. Finally, the remaining family members I-1, II-1, II-4, and III-1 were 21–75 years old, with no history of PJS.

### Genetic test results

In the proband, 123,591 variants were detected. The detailed sequencing information is displayed in Table 2. WES data were filtered based on genomic loci, functional consequences, and allele frequency. A splicing variant in *STK11* was identified in the proband: chromosome 19, position 1,222,983, c.921-1G > C, NM_000455, rs398123406. In addition, other genes associated with cervical cancer, including *MSH6*, were negative. The prediction program MutationTaster indicated that the variant was deleterious (prediction score = 1); however, SIFT and PolyPhen2 did not provide predicted values.

**Table 2:** Whole exome sequencing detail of the proband

Exome Capture Statistics	Proband
Raw reads (bp)	95,647,462
Duplicate (bp)	19,025,592
Mapped (%)	99.71
Properly mapped (%)	99.15
Initial bases on target (bp)	60,456,963
Total effective yield (Mb)	14270.28
Effective yield on target (Mb)	10681.05
Bases covered on target (bp)	60,160,986
Coverage of target region (%)	99.5
Fraction of effective bases on target (%)	74.8
Average sequence depth on target (X)	176.67
Fraction of target region covered >=10X (%)	98.1
Fraction of target region covered >=4X (%)	99.1
Gender	Female

To offer a theoretical basis for genetic counseling, Sanger sequencing was performed to identify whether other family members harbored the c.921-1G > C variant. The results showed that the sister (II-3) and nephew (III-2) of the proband also had the same variant. The other family members did not carry the variant (Fig. 1H/I), which co-segregated with PJS. The variant frequency has not been recorded in any database, but it is classified as pathogenic in the ClinVar database. Therefore, this rare splicing variant was considered a disease-causing variant.

### Splicing defect in *STK11* c.921-1G > C and analysis of the abnormal protein

**Fig. 2:**
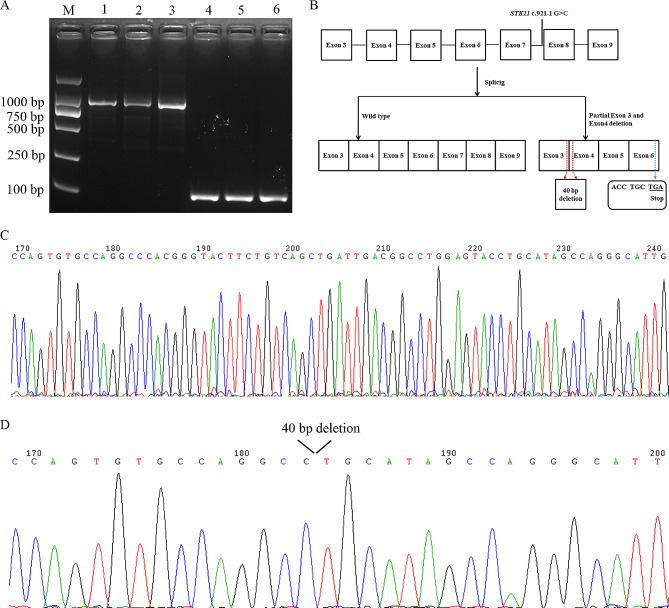
Alternative splicing of *STK11* c.921-1G > C. A: RT-PCR was performed to detect the splicing patterns of *STK11*, with exons 2–9 as the amplified region; M: markers; 1 and 4: healthy control 1; 2 and 5: proband II-2; 3 and 6: healthy control 2, 4, 5, and 6 electrophoresis channels were for the internal control (GAPDH) expression. The expression level of *STK11* mRNA in the proband was < 50% of that in healthy controls. B–C: Sanger sequencing of alternatively spliced products; C) wild type and D) deletion of 40 base pairs between exons 3 and 4. (B) Schematic representation of splicing models

RT-PCR was used to assess the splicing patterns of *STK11*, and exons 2–9 were amplified. The resultant RT-PCR products of the proband and healthy controls were analyzed using gel electrophoresis. An electrophoretic band of approximately 1000 bp (in theory, the amplified band should be 914 bp) was detected after gel electrophoresis (Fig. 2A). However, the sequencing of the RT-PCR products (electrophoretic channel 2, the proband) revealed both a normal transcript (Fig. 2C) and a truncated transcript with 40-nucleotide deletion in *STK11* exons 3 and 4 (Fig. 2D). Meanwhile, only one normal transcript was detected in electrophoretic channels 1 and 3 (914 bp, wild type).

The aberrant transcript (deletion of 40 base pairs between exons 3 and 4) results in a frameshift variant and premature termination of the amino acid in exon 6 and ultimately leads to the loss of its functional domain in the STK11 protein (Fig. 2B). The rare splicing variant c.921-1G > C in intron 7 of *STK11* is generally believed to affect certain bases near intron 7, including the loss or increase of bases in exon 6 or 8. However, exons 3 and 4 are relatively far from intron 7 and no other *STK11* variation was found. Therefore, the possibility of a partial deletion in exons 3 and 4 caused by this variation is extremely low. Meanwhile, RT-PCR showed that compared with healthy controls, the *STK11* mRNA expression level was < 50% in the proband, indicating that this variation may have resulted in insufficient *STK11* mRNA expression in the proband.

## Discussion

The current study results indicated that the rare splicing variant c.921-1G > C in intron 7 of *STK11* may be a pathogenic variant in patients with PJS. The variation may result in insufficient *STK11* mRNA expression in patients with PJS.

PJS is a rare genetic disorder that frequently presents with cancer in multiple organs. Thus, investigating the genetic basis of PJS can directly improve the comprehension of the molecular mechanisms of familial cancers. *STK11* is the primary pathogenic gene associated with PJS, and many studies have determined a link between its variants and increased cancer risk [18, 19]. Mehenni et al. reported that mutations in exon 6 of *LKB1* were associated with increased cancer risk than mutations in other regions of the gene [20]. In addition, Wang et al. analyzed 116 patients with PJS and observed that a variant in exon 7 of *STK11* was associated with 90% of all incidences of gastrointestinal polyps [21]. Furthermore, Salloch et al. studied 16 *STK11* variants in 22 families and demonstrated that truncated *STK11* variants were closely correlated with an increased risk of polyps and cancer as well as with the requirement of more surgical interventions [22]. Taken together, these findings suggested that variants in different sites have a varied effect on cancer risk across tissues and organs.

According to a previous study, gastric cervical lesions are associated with PJS, with the incidence of GAS in PJS being approximately 11–17% [23, 24]. In the present study, a rare case of malignant adenocarcinoma was investigated, which was diagnosed as PJS and histopathologically confirmed as GAS after surgery. According to literature, increasingly more splicing errors are being found in Chinese patients with PJS [25]. Of note, Wangler et al. reported a 13-year-old male diagnosed with PJS who presented with hyperpigmented macules over his lips, buccal mucosa, and fingertips along with polyps in the small intestine. Genetic analysis revealed a heterozygous c.921-1G > T variant in *STK11*; however, he had no family history of PJS [26]. Therefore, the variant may be a de novo variant in the patient. Ylikorkala et al. also reported two patients with PJS who harbored the *STK11* c.921-1G > C variant. Although splicing defects were suspected, no aberrant transcripts were detected using RT-PCR in that study [27]. This may be attributed to a degradation of unstable mRNA via nonsense-mediated decay. Meanwhile, in the present study, a rare splicing variant in *STK11* (c.921-1G > C) was detected in the PJS family. Of note, an aberrant transcript was detected for the first time. This transcript, a deletion of 40 base pairs between exons 3 and 4, results in a frameshift variant and premature termination of the amino acid at exon 6. However, according to literature, the abnormal splicing (*STK11*, c.921-1G > C) may not be caused by this mutation [27]. The decrease in STK11 protein expression caused by this variant may be the primary reason for the occurrence of PJS. Nevertheless, the underlying molecular mechanism of the *STK11* c.921-1G > C variant in PJS remains unclear, and further prospective studies are needed [28]. Of note, for the first time, the current study reported that women in China with this rare *STK11* variant may be at a high risk of developing the rare disease GAS.

Genetic testing is a highly specific and sensitive tool for PJS diagnosis. As patients with PJS have an elevated risk of developing cancer, the early genetic testing and regular monitoring of high-risk patients with PJS are essential [19]. In addition, it is imperative to understand the clinical presentation and early diagnosis of PJS for implementing relevant screening procedures to detect future malignancies early, avoid further complications, and initiate appropriate treatment [29]. For instance, patient III-2 developed melanosis and is recommended to undergo annual gastroscopy to remove gastrointestinal polyps early and delay or prevent cancer development. The study findings suggested that a rare variant (c.921-1G > C) in *STK11* causes PJS, thereby reaffirming the significance of genetic testing in patients with PJS [30].

Taken together, the present study indicated that the rare splicing variant c.921-1G > C in intron 7 of *STK11* may be a pathogenic variant in patients with PJS. However, the variant (in intron 7) may not lead to the production of abnormal transcripts (deletion of 40 base pairs between exons 3 and 4), and PJS may be caused by the decrease in STK11 expression. Of note, this is the first study to report that women in China with this rare *STK11* variant may be at a high risk of developing the rare disease GAS.

### Electronic supplementary material

Below is the link to the electronic supplementary material.

Supplementary Material 1

Supplementary Material 2

## Data Availability

The data hat support the findings of this study are available from the corresponding author upon reasonable request.
