# Supplementary figures and images for: Genetic variation at a splicing branch point in intron 7 of STK11: a rare variant decreasing its expression in a Chinese family with Peutz–Jeghers syndrome
Source: World J Surg Oncol. 2024 Jul 30;22:202. doi: 10.1186/s12957-024-03475-6 (PMC11290102; doi:10.1186/s12957-024-03475-6)

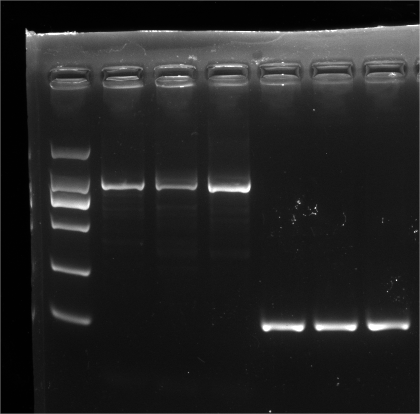

Supplement: Supplementary file 1 — Supplementary Material 1 [file 12957_2024_3475_MOESM1_ESM.png]
