# Supplementary material for: Genetic variation at a splicing branch point in intron 7 of STK11: a rare variant decreasing its expression in a Chinese family with Peutz–Jeghers syndrome
Source: World J Surg Oncol. 2024 Jul 30;22:202. doi: 10.1186/s12957-024-03475-6 (PMC11290102; doi:10.1186/s12957-024-03475-6)
Supplement: Supplementary file 2 — Supplementary Material 2 [file 12957_2024_3475_MOESM2_ESM.pdf]

CERTIFICATE OF ENGLISH EDITING

This is to certify that the manuscript entitled

Genetic variation at a splicing branch point in intron 7 of STK11: a rare variant decreasing its expression in a Chinese family with Peutz–Jeghers syndrome

commissioned to us has been carefully edited by a native English-speaking editor of MogoEdit, and the grammar, spelling, and punctuation have been verified and corrected where needed. Based on this review, we believe that the language in this paper meets academic journal requirements. Please contact us with any questions.

Gang Zhang

Dr. Gang Zhang
Founder & CEO of MogoEdit

Date of Issue
June 24, 2024

Disclaimer: The changes in the document may be accepted or rejected by the authors in their sole discretion after our editing. However, MogoEdit is not responsible for revisions made to the document after our edit on **June 24, 2024**.

MogoEdit is a professional English editing company who provides English language editing, translation, and publication support services to individuals and corporate customers worldwide. As a company invested by the affiliate fund of Chinese Academy of Science, MogoEdit is one of the leading language editing service providers in China, whose clients come from more than 1000 universities and research institutes.
MogoEdit Website: http://www.mogoedit.com/
500+ native English editors: http://www.mogoedit.com/editors

Mogo Internet Technology Co., LTD.
No. 57, 3rd Keji Road, Xi'an 710075, PR China +86 02988317483 support@mogoedit.com
